# Two Cases of Perihepatitis With the Liver Capsule Irritation Sign: A New Physical Examination Technique

**DOI:** 10.7759/cureus.34327

**Published:** 2023-01-29

**Authors:** Mihiro Kaga, Shota Ito, Takeshi Ueda

**Affiliations:** 1 Emergency and General Internal Medicine, Rakuwakai Marutamachi Hospital, Kyoto, JPN; 2 Department of Cardiology, Higashi-Osaka Medical Center, Osaka, JPN

**Keywords:** right upper quadrant abdominal pain, the liver capsule irritation sign, the peritoneal irritation sign, ultrasound (u/s), perihepatitis, fitz-hugh-curtis syndrome

## Abstract

Perihepatitis, including Fitz-Hugh-Curtis syndrome, is an uncommon, chronic manifestation of pelvic inflammatory disease usually affecting premenopausal women. It causes right upper quadrant pain due to inflammation of the liver capsule and adhesion of the peritoneum. Since delayed diagnosis of Fitz-Hugh-Curtis syndrome can lead to infertility and other complications, physical examination findings need to be investigated to predict perihepatitis in the early stages of the disease. Here, we hypothesized that perihepatitis is characterized by increased tenderness and spontaneous pain in the right upper abdomen when the patient is placed in the left lateral recumbent position (we termed this indication the "liver capsule irritation sign"). We examined the patients physically for the presence of this liver capsule irritation sign for an early diagnosis of perihepatitis. We report the first two cases of perihepatitis due to Fitz-Hugh-Curtis syndrome in which the liver capsule irritation sign observed during the physical examination was used for diagnosis. The liver capsule irritation sign is caused by two mechanisms: first, the liver falls gravitationally into the left lateral recumbent position, which makes the liver easier to palpate; and second, the peritoneum is stretched and thus stimulated. The second mechanism is that the transverse colon running around the right upper abdomen slumps gravitationally when the patient is in the left lateral recumbent position, allowing for direct palpation of the liver. The liver capsule irritation sign can be a useful physical finding, suggestive of perihepatitis due to Fitz-Hugh-Curtis syndrome. It may also be suitable in cases of perihepatitis caused by factors other than Fitz-Hugh-Curtis syndrome.

## Introduction

Perihepatitis, often referred to as Fitz-Hugh-Curtis syndrome, is inflammation of the liver capsule with adhesion formation, resulting in right upper quadrant pain [1]. Fitz-Hugh-Curtis syndrome is an uncommon complication of pelvic inflammatory disease. Pelvic inflammatory disease is a sexually transmitted disease, predominantly caused by chlamydia, gonorrhea, or other sexually transmitted infections, that leads to ascending, lymphatic, or hematogenous inflammation [1]. Contrast-enhanced computed tomography (CT) of the abdomen in Fitz-Hugh-Curtis syndrome shows a contrast effect along the liver capsule (mainly from the medial segment to the lateral right lobe) in the early contrast phase, suggesting increased blood flow associated with inflammation extending from the perihepatic region to the liver capsule [2,3]. However, there are no specific physical findings in the abdomen, and it is difficult to differentiate Fitz-Hugh-Curtis syndrome from various other causes of abdominal pain that can cause right upper quadrant pain [4]. This often results in a delayed or missed diagnosis [4].

The diagnosis of Fitz-Hugh-Curtis syndrome can be confirmed by laparoscopy or laparotomy via visualization of adhesions between the diaphragm and liver or the liver and the anterior abdominal wall; still, in most cases, the diagnosis is made comprehensively based on clinical and laboratory findings because of the invasive nature of the procedure [4]. The Centers for Disease Control and Prevention advises the physicians to maintain a low threshold for aggressive treatment because diagnosis of pelvic inflammatory disease is challenging, with a high potential for serious complications [1]. Physical findings that raise the suspicion for perihepatitis early in the physician visit need to be investigated because a delayed diagnosis of Fitz-Hugh-Curtis syndrome can lead to infertility and intestinal obstruction [1,5].

A Japanese study reported enhanced tenderness in the right upper abdomen using an ultrasound probe when a patient with Fitz-Hugh-Curtis syndrome was placed in the left lateral recumbent position, suggesting that this feature may be indicative of perihepatitis [6]. Therefore, we hypothesized that in perihepatitis, the right upper quadrant tenderness and spontaneous pain are elevated when the patient is placed in the left lateral recumbent position (we termed this indication the "liver capsule irritation sign"). We report two cases of perihepatitis due to Fitz-Hugh-Curtis syndrome in which this technique was observed. The purpose of this report is to introduce a method for observation of this sign, discuss the likely mechanisms for it, and validate the signs observed.

## Case presentation

Case 1

**Figure 1:**
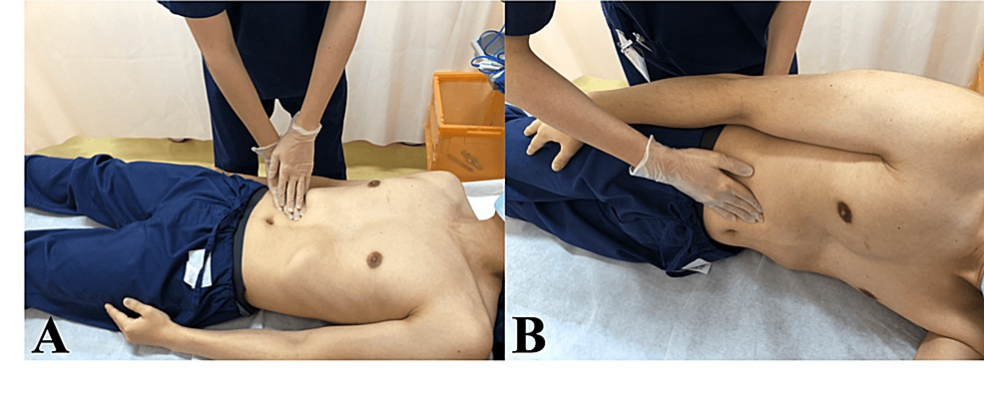
The technique of examination of the liver capsule irritation sign. First, an abdominal examination is performed in the supine position (A). Next, the patient is placed in the left lateral decubitus, and the abdomen is palpated (B). A positive result is obtained when there is increased tenderness and spontaneous pain in the right upper abdomen. This figure depicts the procedure for evaluation of the liver capsule irritation sign in a normal male participant (not any of the patients from this study), to showcase the method employed for the two patients described in this study.

**Figure 2:**
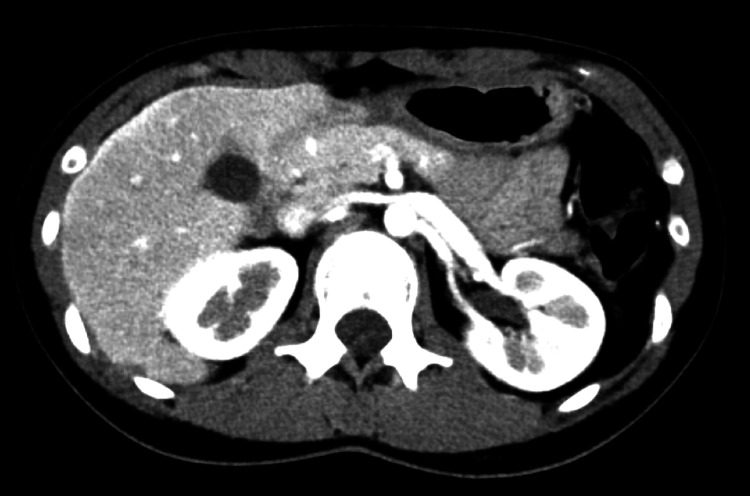
Early phase of contrast-enhanced CT of the abdomen. A contrast effect was observed along the liver capsule, mainly in the right lobe of the liver. CT: computed tomography.

An 18-year-old female patient presented to the emergency department with a main complaint of right upper abdominal pain since the previous three days. She had suffered from lower abdominal pain for several days, one month before her visit, which had resolved spontaneously. She had no past history of either any medication, or of pregnancy and childbirth; however, she had a history of unprotected sexual intercourse. Her vital signs at the time of admission were: body temperature (BT) of 36.6 °C, blood pressure (BP) of 114/79 mmHg, heart rate (HR) of 88 beats/min, respiratory rate of 16 breaths/min, and SpO_2_ (room) of 98%. On physical examination, there were no specific findings in the head, neck, or chest. The abdomen was flat and soft, with tenderness in the right upper quadrant. There was no rebound pain or muscular defense, but the cough sign was positive [7]. When the patient was placed in the left lateral recumbent position and the abdomen was palpated (as per the procedure described in Figure 1), tenderness and spontaneous pain in the right upper abdomen increased (liver capsule irritation sign), which led to the possibility of Fitz-Hugh-Curtis syndrome being considered. Blood tests showed no remarkable findings other than elevated levels of C-reactive protein (CRP) (10.15 mg/dL) and white blood cell count (9400/µL), respectively. Urinalysis showed pyuria and bacteriuria, and she tested positive for the urine polymerase chain reaction test for *Chlamydia trachomatis* DNA. Abdominal contrast-enhanced CT exhibited a contrast effect along the liver capsule in the early contrast-enhanced phase, mainly in the right lobe of the liver (Figure 2). The patient's symptoms subsequently improved with appropriate antimicrobial therapy.

Case 2

**Figure 3:**
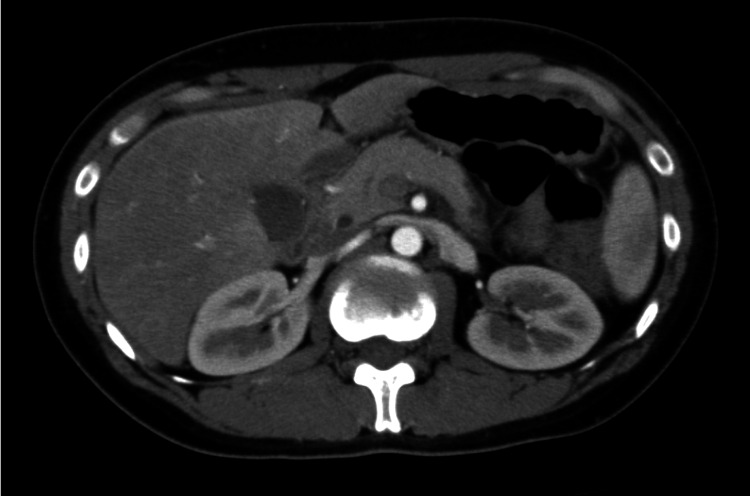
Early phase of contrast-enhanced CT of the abdomen. A contrast effect was observed along the liver capsule, mainly in the right lobe of the liver. CT: computed tomography.

A 25-year-old woman presented to the emergency department with a primary complaint of intermittent epigastric and right upper quadrant pain for the previous two weeks. She had no past history of either any medication or of pregnancy and childbirth, apart from unprotected sexual intercourse. Her vital signs at the time of admission were: BT 36.4 °C, BP 118/62 mmHg, HR 98 beats/min, and SpO_2_ (room) 99%. On physical examination, there were no remarkable findings in the head, neck, or chest. The abdomen was flat and soft, with tenderness in the epigastric region and right upper quadrant. There was rebound pain and percussion tenderness in the right upper abdomen [8]. When the patient was placed in the left lateral recumbent position and the abdomen was palpated (as per the procedure described in Figure 1), tenderness and spontaneous pain increased in the right upper abdomen (liver capsule irritation sign). Since no obvious cause of abdominal pain could be identified by abdominal ultrasound and non-contrast CT, we suspected a peptic ulcer and prescribed lansoprazole 15 mg once daily after breakfast for three days and 10 doses of acetaminophen 500 mg for pain. We decided to follow up with the case as an outpatient. The patient returned to the outpatient clinic the next day with persistent epigastric and right upper abdominal pain. Blood tests revealed elevated levels of CRP (9.56 mg/dL) and white blood cell count (10,900/μL), respectively, but nothing else of note. Urinalysis was positive for urinary *C. trachomatis* DNA. Abdominal contrast-enhanced CT showed a contrast effect along the liver capsule in the early contrast phase, mainly in the right lobe of the liver (Figure 3), and a diagnosis of perihepatitis associated with Fitz-Hugh-Curtis syndrome was confirmed. The patient's symptoms subsequently improved with appropriate antimicrobial therapy.

## Discussion

These two cases are the first reports of perihepatitis due to Fitz-Hugh-Curtis syndrome to be examined with the liver capsule irritation sign on physical examination, which was subsequently confirmed with relevant clinical investigations. We believe that the liver capsule irritation sign can be valuable as a specific physical examination finding indicative of perihepatic inflammation. It has been reported earlier that right upper abdominal tenderness, observed using an ultrasound probe, was enhanced when a patient with Fitz-Hugh-Curtis syndrome was placed in the left lateral recumbent position; however, the mechanism of this enhancement was not discussed [6].

The first mechanism that causes observation of the liver capsule irritation sign in perihepatitis is the gravitational drop of the right lobe of the liver when the body is placed in the left lateral recumbent position. Although the liver is anatomically fixed by several ligaments, it is to some extent a mobile organ [9,10], and surgical and radiological studies have reported that the right lobe of the liver drops in the direction of gravity when the patient is in the left lateral recumbent position [11,12]. Clinical studies using ultrasound have also shown that the left lobe of the liver drops in the direction of gravity when the patient is placed in the left lateral recumbent position, indicating that the liver may compress the inferior vena cava in this position [13-15]. Conversely, it has also been found that the liver slumps in the direction of gravity when the patient is placed in the right lateral recumbent position, indicating that the liver is an organ that tends to incline in the direction of gravity when the body is placed in the lateral recumbent position [16]. For this reason, in patients with perihepatitis, the liver is more easily palpable, and the tenderness intensifies when the patient is placed in the left lateral recumbent position. Moreover, perihepatitis is often observed in the right lobe of the liver on contrast-enhanced CT [2], which may be because inflammatory components such as ascites tend to collect in the right lobe of the liver around Morrison's fossa, the lowest point of the right upper quadrant anatomically [3,15].

When the right lobe of the liver, which is prone to inflammatory findings, is placed in the left lateral recumbent position, two forces act on the liver capsule of the right lobe, one in the direction of gravity and the other in the opposite direction due to adhesion between the liver capsule and visceral peritoneum, causing the liver capsule of the right lobe to stretch. Abdominal pain caused by perihepatitis is a type of visceral pain, which is felt when pain receptors in the peritoneum are stimulated upon stretching [17,18]. This is the reason why spontaneous pain in the right upper abdomen increases in the left lateral recumbent position. In addition, a possible mechanism could be that the ligamentous structures anchored to the liver are pulled by the left lateral recumbency of the gravitationally inclining liver, thereby stretching the hepatic capsule around the ligament and aggravating the spontaneous pain. An example of an already-known examination technique that applies the principle that positional changes can shift the position of organs and aggravate painful stimuli is Rosenstein's sign, which is seen in appendicitis. Rosenstein's sign is a symptom of increased pain when pressure is applied to McBurney's point in the left lateral recumbent position compared to the supine position and is thought to be caused by tension on the mesoappendix since the appendix is stretched by the gravitational drop of the ileocecum. Rosenstein's sign, upon physical examination, can detect localized inflammation around the appendix. However, in this case, the liver capsule irritation sign was able to detect inflammation around Morrison's fossa, which could not be palpated directly. Since Morrison's fossa cannot be directly palpated, it has been considered difficult to determine peritonitis confined to this area by physical examination, which the liver capsule irritation sign was able to detect.

Another mechanism that is responsible for the observation of the liver capsule irritation sign in perihepatitis is that the transverse colon running around the right upper abdomen drops in the direction of gravity when the patient is in the left lateral recumbent position. Since the transverse colon is not anchored to the retroperitoneum, the left lateral recumbency causes the transverse colon to slump in the direction of gravity, allowing direct contact with the liver when the right upper abdomen is palpated, which may exacerbate right upper abdominal tenderness [10,19]. These two mechanisms would suggest that having a patient with perihepatitis placed in the left lateral recumbent position and palpating the abdomen would exacerbate the tenderness and spontaneous pain in the right upper abdomen.

The findings of the contrast effect along the liver capsule in the early phase of contrast-enhanced CT are helpful in diagnosing perihepatitis, which can be seen not only in Fitz-Hugh-Curtis syndrome but also in other conditions such as systemic lupus erythematosus, liver abscess, cholangitis, peritonitis due to cancer, tuberculosis, or other causes, acute cholecystitis, superior vena cava obstruction, and congenital hepatic fibrosis [2]. The liver capsule irritation sign may be found in various inflammatory diseases, such as collagen disease and intra-abdominal infections, and the sign may be a useful diagnostic parameter for inflammatory diseases other than Fitz-Hugh-Curtis syndrome. Thus, the liver capsule irritation sign is a useful physical finding as one of the peritoneal irritation signs, along with the cough sign and percussion tenderness, as observed in this case. In the future, more case reports demonstrating the usefulness of this sign for diagnosing perihepatitis other than Fitz-Hugh-Curtis syndrome will be needed [7,8], to determine the likelihood ratio of this sign.

## Conclusions

The liver capsule irritation sign is a useful physical examination finding that is indicative of perihepatitis due to Fitz-Hugh-Curtis syndrome in emergency room clinical settings. This sign may also be useful in patients with perihepatitis due to causes other than Fitz-Hugh-Curtis syndrome who present with right upper quadrant pain. Thus, the liver capsule irritation sign on physical examination can help in the differential diagnosis prior to the laboratory clinical examinations.
